# Transcriptome profiles of latently- and reactivated HIV-1 infected primary CD4^+^ T cells: A pooled data-analysis

**DOI:** 10.3389/fimmu.2022.915805

**Published:** 2022-08-26

**Authors:** Anne Inderbitzin, Tom Loosli, Lennart Opitz, Peter Rusert, Karin J. Metzner

**Affiliations:** ^1^ Department of Infectious Diseases and Hospital Epidemiology, University Hospital Zurich, Zurich, Switzerland; ^2^ Institute of Medical Virology, University of Zurich, Zurich, Switzerland; ^3^ Life Science Zurich Graduate School, University of Zurich, Zurich, Switzerland; ^4^ Functional Genomics Center Zurich, Eidgenössische Technische Hochschule (ETH) Zürich/University of Zurich, Zurich, Switzerland

**Keywords:** latently HIV-1 infected primary CD4^+^ T cells, reactivated HIV-1 infected primary CD4^+^ T cells, HIV-1 latency reversal agents, transcriptome profile, primary CD4^+^ T cell models of HIV-1 latency, pooled data-analysis, pooled data-analysis differentially expressed genes (pdaDEGs)

## Abstract

The main obstacle to cure HIV-1 is the latent reservoir. Antiretroviral therapy effectively controls viral replication, however, it does not eradicate the latent reservoir. Latent CD4^+^ T cells are extremely rare in HIV-1 infected patients, making primary CD4^+^ T cell models of HIV-1 latency key to understanding latency and thus finding a cure. In recent years several primary CD4^+^ T cell models of HIV-1 latency were developed to study the underlying mechanism of establishing, maintaining and reversing HIV-1 latency. In the search of biomarkers, primary CD4^+^ T cell models of HIV-1 latency were used for bulk and single-cell transcriptomics. A wealth of information was generated from transcriptome analyses of different primary CD4^+^ T cell models of HIV-1 latency using latently- and reactivated HIV-1 infected primary CD4^+^ T cells. Here, we performed a pooled data-analysis comparing the transcriptome profiles of latently- and reactivated HIV-1 infected cells of 5 *in vitro* primary CD4^+^ T cell models of HIV-1 latency and 2 *ex vivo* studies of reactivated HIV-1 infected primary CD4^+^ T cells from HIV-1 infected individuals. Identifying genes that are differentially expressed between latently- and reactivated HIV-1 infected primary CD4^+^ T cells could be a more successful strategy to better understand and characterize HIV-1 latency and reactivation. We observed that natural ligands and coreceptors were predominantly downregulated in latently HIV-1 infected primary CD4^+^ T cells, whereas genes associated with apoptosis, cell cycle and HLA class II were upregulated in reactivated HIV-1 infected primary CD4^+^ T cells. In addition, we observed 5 differentially expressed genes that co-occurred in latently- and reactivated HIV-1 infected primary CD4^+^ T cells, one of which, MSRB2, was found to be differentially expressed between latently- and reactivated HIV-1 infected cells. Investigation of primary CD4^+^ T cell models of HIV-1 latency that mimic the *in vivo* state remains essential for the study of HIV-1 latency and thus providing the opportunity to compare the transcriptome profile of latently- and reactivated HIV-1 infected cells to gain insights into differentially expressed genes, which might contribute to HIV-1 latency.

## Introduction

The human immunodeficiency virus type 1 (HIV-1) remains a global health problem, while ART efficiently blocks viral replication it does not cure HIV-1 infection owing to persistent proviruses (1). These proviruses are quiescent and mainly found in resting memory CD4^+^ T cells, known as the latent reservoir (2, 3). The latent reservoir is defined as replication-competent but transcriptionally silent viruses. The latent reservoir is established within the first weeks of infection (4, 5) but the exact mechanisms of its establishment is still being investigated. Potential mechanisms leading HIV-1 into latency include transcriptional interference, chromatin remodelling, epigenetic silencing, and transcription factor sequestration (6). Nevertheless, to date the driving forces for HIV-1 latency are not fully understood. It is still unknown which factors distinguish between latently- and reactivated HIV-1 infected CD4^+^ T cells on a molecular level. Numerous studies searched for cellular markers identifying latently HIV-1 infected cells and several cellular markers were described, however, these cellular markers could only rarely be confirmed and are controversially discussed (7).

Therefore, primary CD4^+^ T cell models of HIV-1 latency that mimic the *in vivo* state remain a necessity for the study of HIV-1 latency. A wealth of information has been generated from the transcriptome profiles of primary CD4^+^ T cell models of HIV-1 latency. To obtain a comprehensive understanding of drivers that might maintain HIV-1 in latency, we performed a pooled data-analysis comparing the transcriptome profiles of latently- and reactivated HIV-1 infected cells from 5 *in vitro* primary CD4^+^ T cell models of HIV-1 latency and 2 *ex vivo* studies of reactivated HIV-1 infected primary CD4^+^ T cells from HIV-1 infected individuals (detailed description of the models/studies in **Supplementary Figure 1** and **Supplementary Material**). By conducting a pooled data-analysis, high-throughput data from multiple independent primary CD4^+^ T cell models of HIV-1 latency are included, resulting in 1. larger sample size, 2. overcoming donor variability bias and 3. allowing for a comprehensive assessment of transcriptome profiles, thus providing more insights into HIV-1 pathogenesis and latency. In our pooled data-analysis, we identified 247 differentially expressed genes (DEGs) that were present in at least 3 of 4-5 datasets of latently- and reactivated HIV-1-infected primary CD4^+^ T cells, respectively. These DEGs were called pooled data-analysis differentially expressed genes (pdaDEGs). This may be a successful strategy to better understand and characterize HIV-1 latency and reactivation. This could provide insights into the mechanisms leading to HIV-1 latency and reactivation.

## Results

### Quantitative assessment of gene expression from data sets of *in vitro* primary CD4^+^ T cell models of HIV-1 latency and *ex vivo* studies of reactivated HIV-1 infected primary CD4^+^ T cells from HIV-1 infected individuals

**Figure 1:**
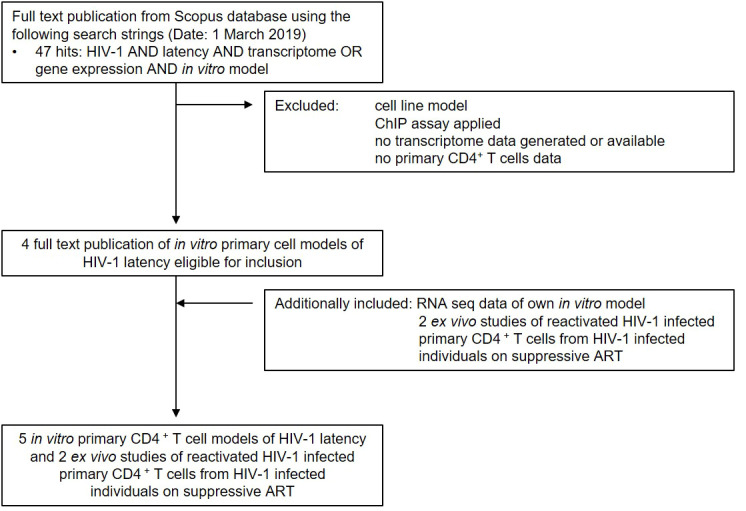
Summary of study search and selection procedure from the Scopus database.

To gain a comprehensive understanding of differentially expressed genes (DEGs) between latently- and reactivated HIV-1 infected cells, we analysed DEGs in 5 *in vitro* primary CD4^+^ T cell models of HIV-1 latency and 2 *ex vivo* studies of reactivated HIV-1 infected primary CD4^+^ T cells from HIV-1 infected individuals, in particular latently- (8–11) and reactivated (10–13) HIV-1 infected cells. Applying our search parameters resulted in 47 full text publications. We excluded studies using ChIP assays, non-primary CD4^+^ T cells data, cell line studies, and studies which did not contain transcriptome data and resulted in 4 *in vitro* primary CD4^+^ T cell models of HIV-1 latency (8–11) (**Figure 1**). By including 2 *ex vivo* studies of reactivated HIV-1 infected primary CD4^+^ T cells from suppressed HIV-1 infected individuals and our own model (14), a total of 50 unique transcriptome samples (26 HIV-1 infected cells and 24 uninfected cells) were included in our pooled data-analysis. We obtained 1’297-21’886 HGNC annotated genes per dataset (**Table 1**). We determined the mean standardised expression of all genes in all primary CD4^+^ T cell models of HIV-1 latency by averaging the fold changes standardized by the study-specific standard error. To account for the differences in the datasets, we refrained from identifying the DEGs by |fold change| >2 and false discovery rate (FDR) <0.05, but employed a custom filtering score method based on: 1. FDR <0.1 increasing the score by 1, 2. absolute fold change greater than the study-specific 50% absolute fold change quantile, increasing the score by 1, and 3. up- or downregulation, resulting in either a positive or negative score, respectively. A gene can get a score of maximally 2 per dataset, i.e., maximum score of 8 for latently and 10 for reactivated HIV-1 infected cells (**Table 1**). For each gene the scores are summed up across datasets and normalized to 1. Pooled data-analysis DEGs (pdaDEGs) were then identified by applying a filtering score ≥0.5. This resulted in 130 pdaDEGs for latently- and 117 pdaDEGs for reactivated HIV-1 infected primary CD4^+^ T cells (**Table 1**). The 130 pdaDEGs obtained from the latently HIV-1 infected cells were present in at least 3 out of the 4 primary CD4^+^ T cell models of HIV-1 latency. Whereas in the reactivated HIV-1 infected cells 117 pdaDEGs were present in at least 3 out of 5 datasets. In general, pdaDEGs were more frequently downregulated in latently HIV-1 infected primary CD4^+^ T cells and more frequently upregulated in reactivated HIV-1 infected primary CD4^+^ T cells (**Table 1**).

**Table 1:** Cross-reference of 5 *in vitro* primary CD4^+^ T cell models of HIV-1 latency and 2 *ex vivo* studies of reactivated HIV-1 infected primary CD4^+^ T cells from HIV-1 infected individuals, of latently- and reactivated HIV-1 infected cells.

Study	Dataset	Gene IDs of dataset*	Gene IDs after filtering**	HGNC annotated genes	pdaDEGs***	Up and downregulated pdaDEGs***
Iglesias-Ussel et al. (9)	latent	1297	1297	1297	104 of 130	up: 45
						down: 59
	latent	50312	15781	13907	121 of 130	up: 52
White et al. (11)						down: 69
	reactivated	50312	16452	14720	112 of 117	up: 71
						down: 41
	latent	50249	21972	21886	129 of 130	up: 62
Mohammadi et al. (10)						down: 67
	reactivated	50249	21051	20972	114 of 117	up: 70
						down: 44
Bradley et al. (8)	latent	21386	17029	16141	128 of 130	up: 51
						down: 77
Inderbitzin et al. (14)	reactivated	21505	14664	13946	109 of 117	up: 65
						down: 44
Cohn et al. (12)	reactivated	28079	11903	11869	116 of 117	up: 82
						down: 34
Kulpa et al. (13)	reactivated	35797	19245	19206	98 of 117	up: 70
						down: 28

* Includes: Ensembl Gene IDs, Hugo Gene Nomenclature Committee (HGNC) symbols, transcript names.

** Removal of non-informative reads and low read count. Includes: Ensembl Gene IDs, Hugo Gene Nomenclature Committee (HGNC) symbols, transcript names.

*** Defined as passing the filter score; 130 and 117 pdaDEGs obtained from datasets of latently- and reactivated HIV-1 infected primary CD4^+^ T cells, respectively. Depicted Gene IDs of dataset, prior and after filtering by score, and up and downregulated pooled data-analysis differentially expressed genes (pdaDEGs).

### pdaDEGs identified in latently HIV-1 infected cells from 4 *in vitro* primary CD4^+^ T cell models of HIV-1 latency

**Figure 2:**
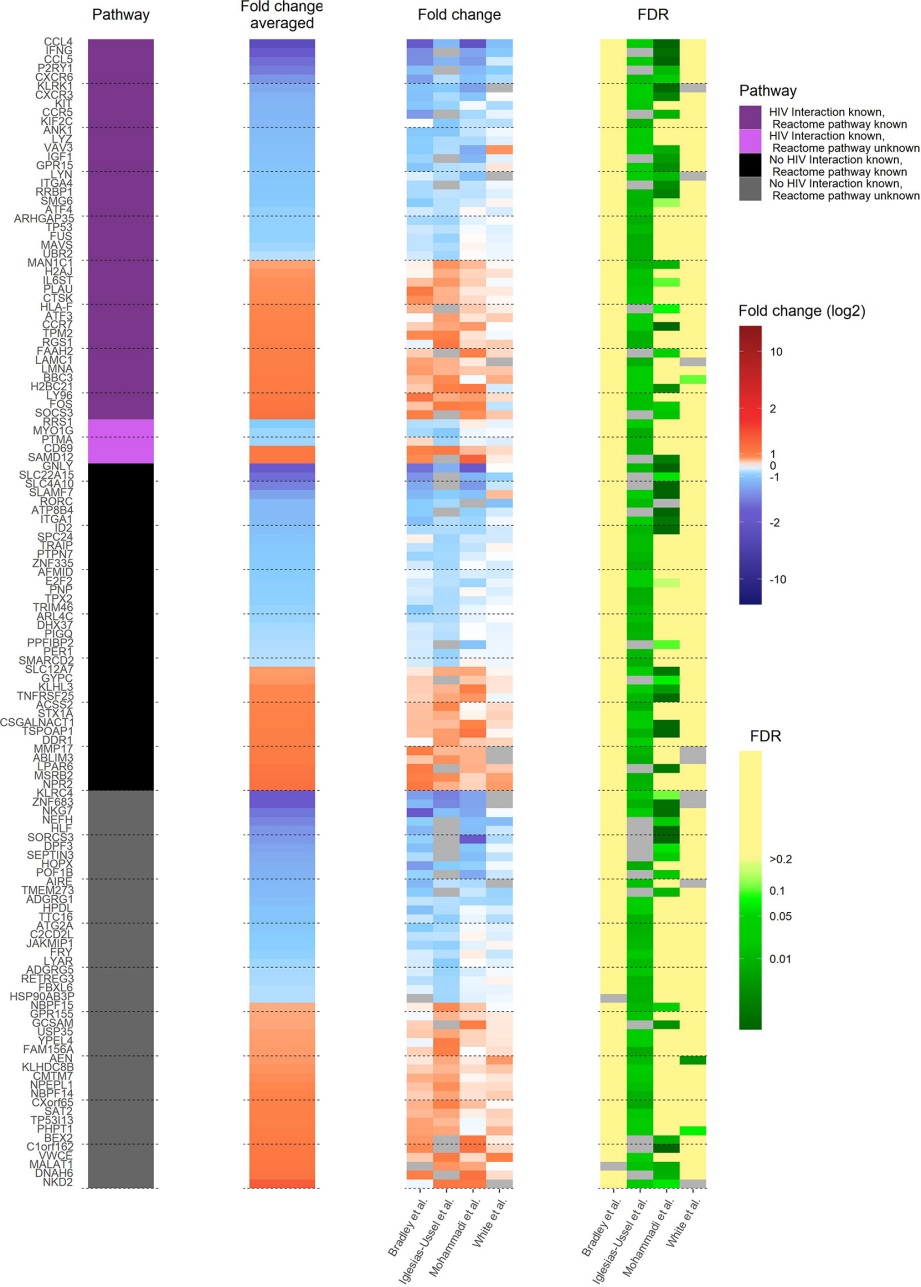
Heat map of transcriptome profile of latently HIV-1 infected cells of 4 *in vitro* primary CD4^+^ T cell models of HIV-1 latency. The 130 pdaDEGs depicted in the heat map are co-occurring in the latently HIV-1 infected cells of the at least 3 of 4 primary CD4^+^ T models of HIV-1 latency. For each gene the available information on pathways, mean standardized fold change, and study-specific fold change and false discovery rates (FDR) are illustrated. The pathway describes whether an HIV-1 interaction/association and/or the reactome pathway is known or not. Fold change and FDR in grey indicates no gene expression reported in the according dataset [Bradley et al. (8) Iglesias-Ussel et al. (9), Mohammadi et al. (10) and White et al. (11)].

A comprehensive understanding of how latently HIV-1 infected primary CD4^+^ T cells are altered to allow HIV-1 persistence would be an important step towards developing cures for HIV-1-infected individuals. Therefore, it is important to determine whether the observed transcriptional heterogeneity in latently HIV-1 infected primary CD4^+^ T cells, which suggests that latent HIV-1 infection can persist in very different host cell environments, indeed masks common core motifs that would be responsible for controlling HIV-1 latency (12, 15, 16). To address this goal, we analysed and compared the 130 identified pdaDEGs in latently HIV-1 infected cells of 4 *in vitro* primary CD4^+^ T cell models of HIV-1 latency (8–11) (**Table 1** and **Supplementary Figure 1A**). Of those 130 pdaDEGs, 75 were down- and 55 upregulated. 48 pdaDEGs showed associations with HIV-1 based on the Database for Annotation, Visualization and Integrated Discovery (DAVID), of which 29/48 were downregulated (**Figure 2**). 9 pdaDEGs with known HIV-1 associations were observed across all 4 *in vitro* primary CD4^+^ T cell models of HIV-1 latency. Out of which, 5 were downregulated (CCL4, CCL5, CXCR6, LYZ and RRBP1) and 4 upregulated (PLAU, LMNA, LY96 and CD69) (**Supplementary Table 1**). Downregulated pdaDEGs were predominantly natural ligands or coreceptor: CCL4 (chemokine (C-C motif) ligands 4), CCL5 (RANTES, regulated on activation, normal T cell expressed and secreted), and CXCR6 (C-X-C chemokine receptor type 6). CCL4 is known to activate and enhance the cytotoxicity in natural killer cells (17). CCL5 has been shown to interfere with the spread of HIV-1 by 1. binding to the CCR5 receptor and thereby blocking the binding of the HIV-1 envelope or 2. inducing the internalisation of the bound receptor and thereby reducing the surface amounts of CCR5 (18–21). CXCR6 was found to be downregulated across primary cell models of HIV-1 latency; it is known as a minor coreceptor of HIV-1 and might play a role in disease progression through its role as mediator of inflammation (22). The main HIV-1 co-receptor CCR5 was also found to be downregulated in 3/4 *in vitro* primary cell models of HIV-1 latency, which is in line with the study from Shan et al., showing that CCR5 is downregulated in resting CD4^+^ T cells (23). In summary, we found that natural ligands and coreceptors were predominantly downregulated in all investigated models for latently HIV-1 infected primary CD4^+^ T cells.

### pdaDEGs identified in reactivated HIV-1 infected cells from 3 *in vitro* primary CD4^+^ T cell models of HIV-1 latency and 2 *ex vivo* studies of reactivated HIV-1 infected primary CD4^+^ T cells from HIV-1 infected individuals

In *in vivo* and *in vitro* settings, a large number of latently HIV-1 infected primary CD4^+^ T cells remain unresponsive to strong latency reversal agents (24–35). To investigate transcriptional heterogeneity in HIV-1 latency reversal and to find common core motifs responsible for controlling HIV-1 latency reactivation, we analysed and compared pdaDEGs in reactivated HIV-1 infected cells of 3 *in vitro* (10, 11, 14) primary CD4^+^ T cell models of HIV-1 latency and 2 *ex vivo s*tudies of reactivated HIV-1 infected primary CD4^+^ T cells from HIV-1 infected individuals (**Supplementary Figure 1**) (12, 13).

**Figure 3:**
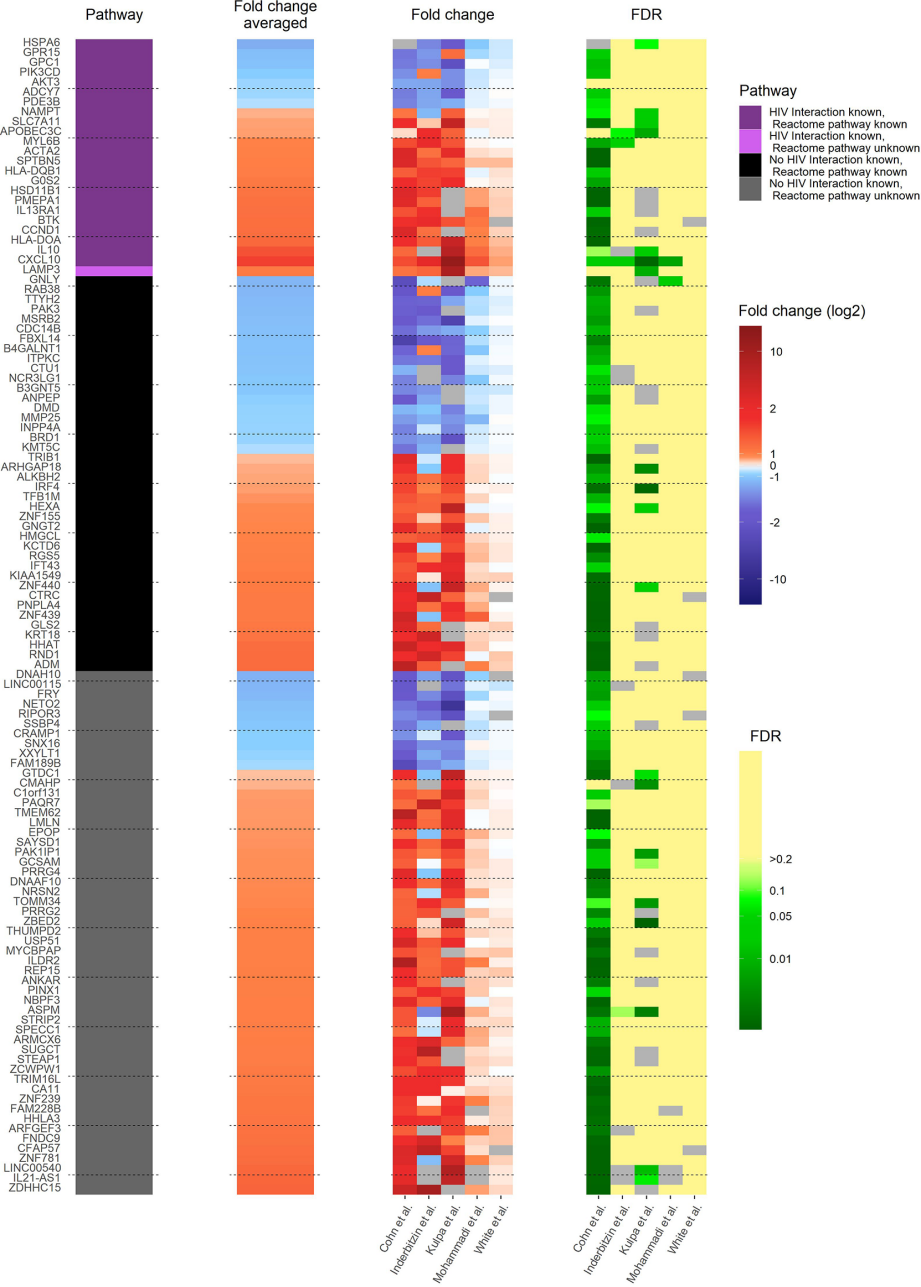
Heat map of gene expression profile of reactivated HIV-1 infected cells of 3 *in vitro* primary CD4^+^ T cell models of HIV-1 latency and 2 *ex vivo* studies of reactivated HIV-1 infected primary CD4^+^ T cells from HIV-1 infected individuals. The 117 pdaDEGs depicted in the heat map co-occurring in the reactivated HIV-1 infected cells of at least 3 out of 5 primary CD4^+^ T cell models of HIV-1 latency. For each gene the available information on pathways, mean standardized fold change, and study-specific fold change and false discovery rates (FDR) are illustrated. The pathway describes whether an HIV-1 interaction/association and/or the reactome pathway is known or not. Fold change and FDR in grey indicates no gene expression reported in the according dataset [Cohn et al. (12), Inderbitzin et al. (14), Kulpa et al. (13), Mohammadi et al. (10) and White et al. (11)].

In the reactivated HIV-1 infected primary CD4^+^ T cells, 117 pdaDEGs were identified, of which 35 pdaDEGs were down-, and 82 upregulated. 24 pdaDEGs have known HIV-1 associations based on DAVID, of which 16/24 were upregulated (**Figure 3**). 6 of those upregulated pdaDEGs with known HIV-1 associations were observed in all 5 datasets, namely ACTA2, LAMP3, HLA-DOA, CXCL10, SLC7A11 and SPTBN5 (**Supplementary Table 2**).

The upregulated pdaDEGs were predominantly genes associated with 1. the p53 pathway, such as ACTA2 (actin alpha 2) which is known to be induced by p53 (11), 2. the PI3K/Akt pathway, such as LAMP3 (lysosome-associated membrane glycoprotein 3) which is involved in cell cycle and apoptosis (36) or 3. the human leukocyte antigen (HLA) class II such as HLA-DOA and HLA-DQB1, which was found in 4/5 *in vitro* and *ex vivo* HIV-1 studies, the latter is associated with increased risk of susceptibility to HIV-1 infection and for a rapid HIV-1 disease progression (37). CXCL10 (C-X-C motif chemokine ligand 10) is a pro-inflammatory cytokine involved in processes such as differentiation, regulation of cell growth, activation of peripheral immune cells and apoptosis (38). In addition, CXCL10 was suggested as a biomarker in the clinic for long-term HIV disease prognosis (39). SLC7A11 (solute carrier family 7 member 11), also named xCT, is a cytoplasmic membrane antiporter that exports one glutamate molecule for each imported cystine molecule (40–43). SLC7A11 has shown to inhibit HIV-1 infection (44).

In summary, we found genes associated with apoptosis, cell cycle and HLA class II were predominantly upregulated in all investigated models for reactivated HIV-1 infected primary CD4^+^ T cells.

### Comparison of transcriptome profiles of latently- and reactivated HIV-1 infected CD4^+^ T cells

**Figure 4:**
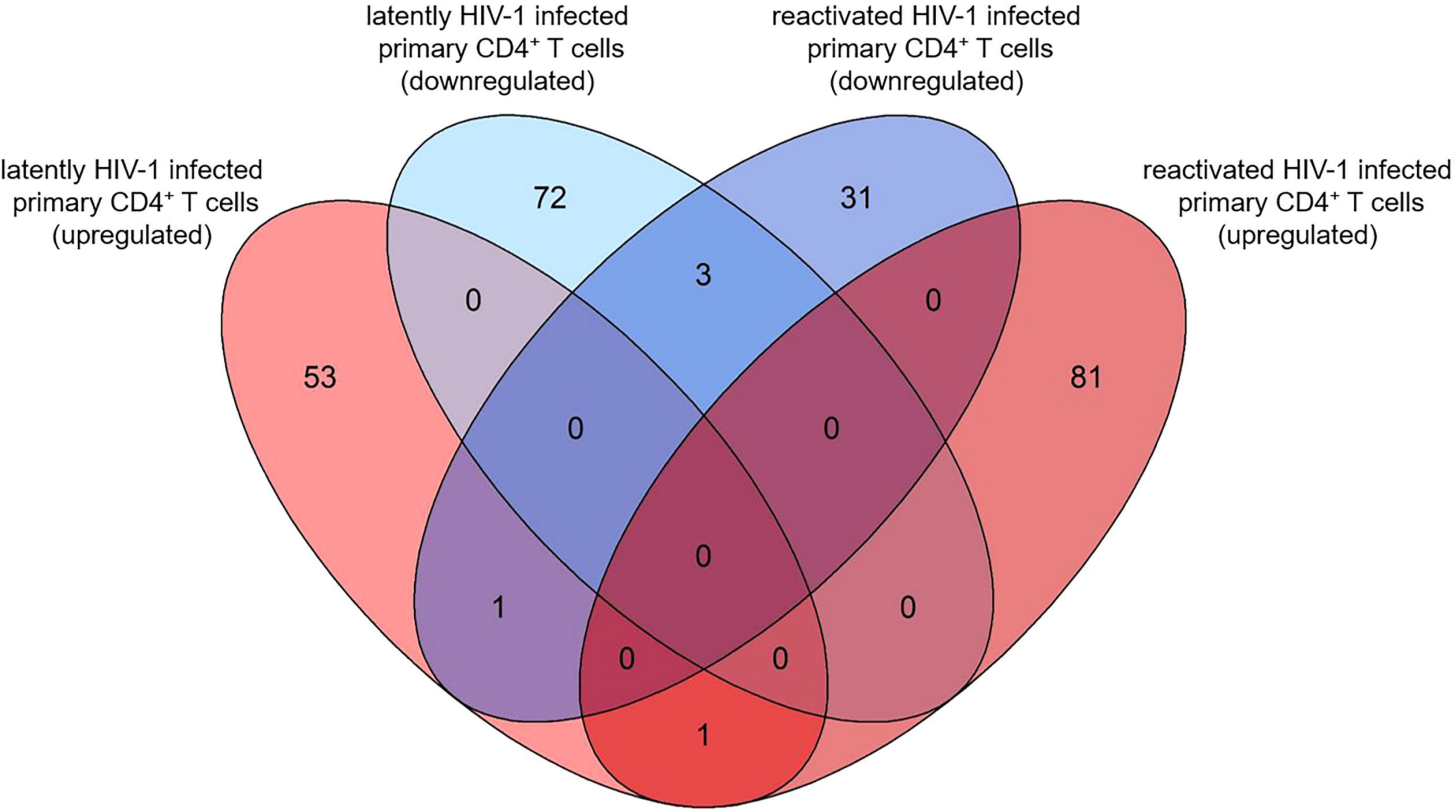
Overlap of pdaDEGs identified in latently- and reactivated HIV-1 infected cells from 5 *in vitro* primary CD4^+^ T cell models of HIV-1 latency and 2 *ex vivo* studies of reactivated HIV-1 infected primary CD4^+^ T cells from HIV-1 infected individuals. Of the 130 and 117 pdaDEGs identified in latently- and reactivated HIV-1 infected cells, respectively, 5 co-occurred in both groups. Of those, 3 pdaDEGs were downregulated, one pdaDEGs upregulated and one pdaDEGs was differentially expressed in latently- and reactivated HIV-1 infected primary CD4^+^ T cells.

Next, we analysed the overlaps of the 130 and 117 pdaDEGs in latently- and reactivated HIV-1 infected primary CD4^+^ T cells, respectively. 5 pdaDEGs were observed in latently- and reactivated HIV-1 infected primary CD4^+^ T cells: FRY, GCSAM, GNLY, GPR15 and MSRB2 (**Figure 4**). Of those pdaDEGs, FRY, GNLY and GPR15 were downregulated and GCSM was upregulated in both groups. Only methionine sulfoxide reductase B2 (MSRB2) was regulated differentially between the latently- and reactivated HIV-1 infected CD4^+^ T cells, namely, upregulated in latently HIV-1 infected primary CD4^+^ T cells and downregulated in reactivated HIV-1 infected primary CD4^+^ T cells. Based on DAVID there is only an HIV-1 interaction known for GPR15, namely an interaction with HIV-1 Env gp120. GPR15 has been found to be the co-receptor of SIV and HIV-2 (45). According to the reactome, FRY and GCSAM could not be assigned to any pathway (**Figures 2**, **3**). All those genes might be interesting in further studies, in particular MSRB2.

### Enriched biological processes of pdaDEGs

**Figure 5:**
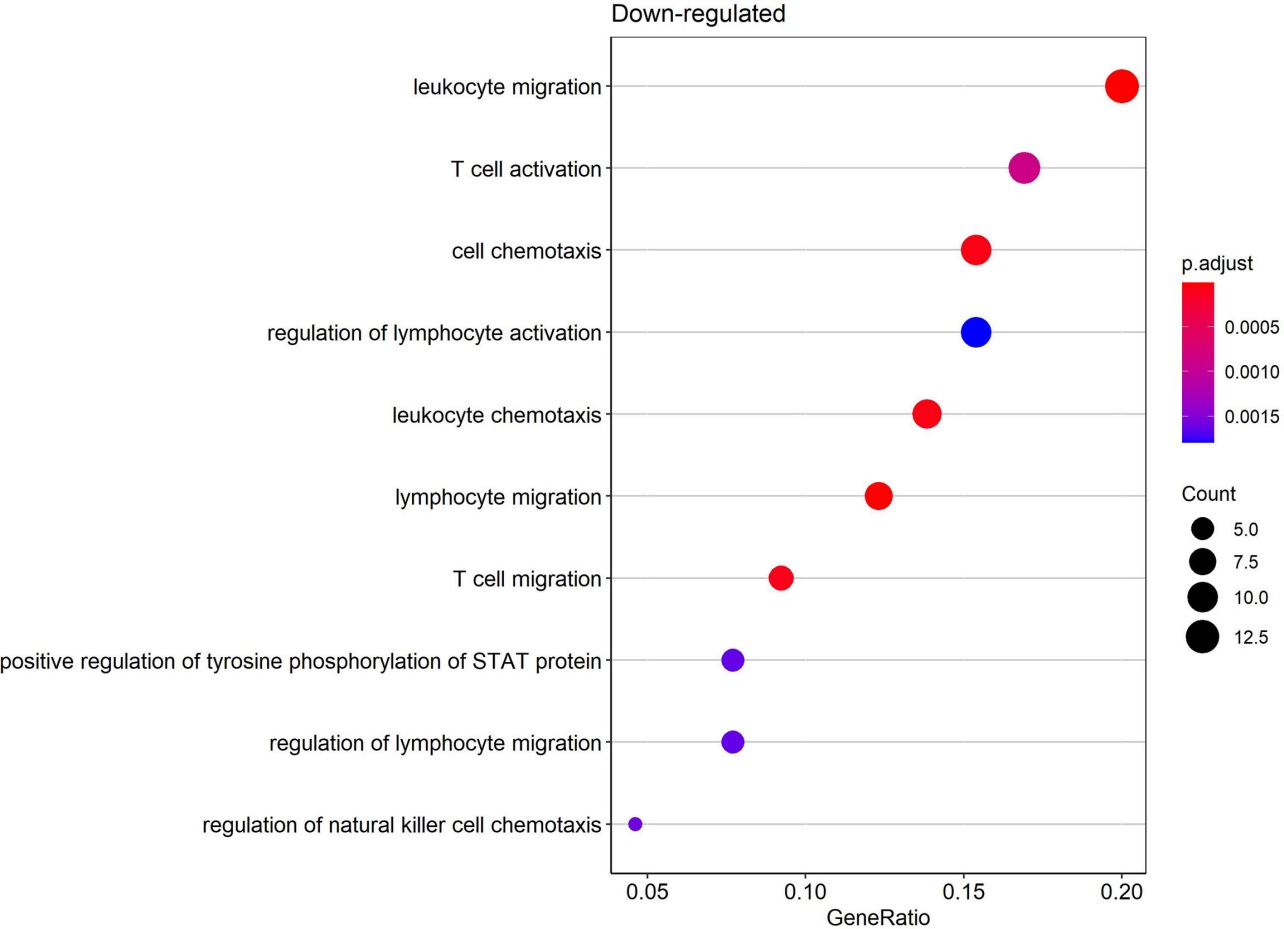
Enriched biological processes by gene ontology (GO) enrichment analysis of downregulated pdaDEGs in latently HIV-1 infected primary CD4^+^ T cells. Depicted are overrepresented biological processes of downregulated pdaDEGs shown by the gene count in circle size and color coded by adjusted p-value.

Gene ontology (GO) enrichment analysis was performed for functional investigation of pdaDEGs. In the GO enrichment analysis we observed predominantly downregulation of the T cell activation and STAT, JAK pathways in latently HIV-1 infected cells of the 4 *in vitro* primary CD4^+^ T cell models of HIV-1 latency (**Figure 5**). In addition, *bona fide* markers for IFN related genes were identified to be downregulated in 3/4 *in vitro* primary CD4^+^ T cell models of HIV-1 latency, such as MAVS, IFNG, TRIM46. In parallel, genes associated with the p53 signalling pathways were found to be upregulated in particular pathways related to apoptosis and DNA damage repair (11), such as BBC3 and TNFRSF25. For the reactivated HIV-1 infected primary CD4^+^ T cells, we could not observe any enriched biological processes in the pdaDEGs.

## Discussion

Current single-cell RNAseq studies of latently HIV-1 infected primary CD4^+^ T cells revealed a high degree of heterogeneity between individual latently HIV-1-infected cells, suggesting that HIV-1 latency can persist in very different host cell environments (12, 15, 16). Host cell heterogeneity may explain, at least in part, the differential responsiveness of latently infected primary CD4^+^ T cells to reactivation. Identifying genes that drive and maintain HIV-1 latency is important to improve current curative strategies. In this study, we performed a pooled data-analysis of transcriptome datasets of latently- and reactivated HIV-1 infected cells from 5 *in vitro* primary CD4^+^ T cell models of HIV-1 latency and 2 *ex vivo* studies of reactivated HIV-1 infected primary CD4^+^ T cells from HIV-1 infected individuals. Although the experimental settings are unique for each *in vitro* primary CD4^+^ T cell models of HIV-1 latency and *ex vivo* studies of reactivated HIV-1 infected primary CD4^+^ T cells from HIV-1 infected individuals, regarding 1. how long cells are maintained in culture prior to infection 2. how CD4^+^ T cell survival is ensured, 3. how reactivation was achieved and 4. using replication competent or incompetent HIV-1, we observed pdaDEGs across *in vitro* primary CD4^+^ T cell models of HIV-1 latency and *ex vivo* studies of reactivated HIV-1 infected primary CD4^+^ T cells from HIV-1 infected individuals. In latently- and reactivated HIV-1 infected primary CD4^+^ T cells we could observe five co-occurring pdaDEGs, one of which MSRB2 was differentially expressed. We observed that natural ligands and coreceptors were predominantly downregulated in latently HIV-1 infected primary CD4^+^ T cells, whereas genes associated with apoptosis, cell cycle and HLA class II were upregulated in reactivated HIV-1 infected CD4^+^ T cells.

Latently HIV-1 infected primary CD4^+^ T cells are rare and to this day no unique markers for their identification is known (46, 47). There are numerous RNAseq/microarrays datasets of HIV-1 infected primary CD4^+^ T cells, which either apply strict fold change >2 and FDR <0.05 filters or select a single candidate to propose as newly discovered HIV-1 latency marker, yet the respective results have rarely been confirmed by others. In this study we combined RNAseq datasets from published primary CD4^+^ T cell models of HIV-1 latency and *ex vivo* studies of reactivated HIV-1 infected primary CD4^+^ T cells from HIV-1 infected individuals, to get a more comprehensive picture. By considering the expression patterns of different datasets, we can identify genes with small effects that would otherwise be missed. Furthermore, we argue that if genes, even if the effect is small, are similarly differentially expressed in different datasets, the effect of HIV-1 infection on dysregulation is even more likely to be.

Among the 130 pdaDEGs in latently- and 117 pdaDEGs in reactivated HIV-1 infected primary CD4^+^ T cells we identified 5 pdaDEGs which co-occurred in both groups. One of which, MSRB2, was differentially expressed, namely upregulated in latently HIV-1 infected primary CD4^+^ T cells and downregulated in reactivated HIV-1 infected primary CD4^+^ T cells. To date, there is no HIV-1 interaction with MSRB2 reported. MSRB2 has been associated with diabetes mellitus (48), Parkinsons’ disease (48) and Alzheimer (49) and MSRB2 has shown to reside in the mitochondrial matrix (50–52). In Parkinson’s disease it was observed to be necessary for induction of mitophagy, a process in which damaged, toxic mitochondria are removed to protect a cell from apoptosis (53, 54). In the absence of MSRB2 it was observed that mitochondria undergo oxidative stress, leading to reduced mitophagy (48). Other studies also made similar observations: MSRB2 protects cell damage from oxidative stress in various cells such as lymphoblast and leukemia cells (55, 56). Therefore, upregulation of MSRB2 in latently HIV-1 infected primary CD4^+^ T cells could inhibit apoptosis, while downregulation in reactivated HIV-1 infected genes leads to apoptosis due to the cytotoxic response of LRA or infectious virus particle release. However, this process has not yet been documented in CD4^+^ T cells and therefore needs further investigation.

Many identified pdaDEGs in latently HIV-1 infected primary CD4^+^ T cells have been reported as *bona fide* markers for antiviral defense and apoptosis, such as IFN related genes or genes associated with the p53 pathway. In reactivated HIV-1 infected primary CD4^+^ T cells we observed upregulation of genes associated with apoptosis, cell cycle and HLA class II, particularly in association with the p53 pathway and the PI3K/Akt pathway. The observed genes can be used in future primary CD4^+^ T cell models of HIV-1 latency to clarify the functional and physiological significance in primary CD4^+^ T cells.

Different LRA were used for reactivation in the *in vitro* primary CD4^+^ T cell models of HIV-1 latency compared to the *ex vivo* studies of reactivated HIV-1 infected primary CD4^+^ T cells from HIV-1 infected individuals, nevertheless we could observe genes to be similarly differentially expressed across studies. However, the impact of differences in clonality of the reservoir exposed to different antiretroviral drugs and LRA treatments could bias transcriptome profiles. We have tried not to become too speculative when comparing latently- and reactivated HIV-1 infected primary CD4^+^ T cells, and have therefore selected genes and pathways already known to play a role in the life cycle and pathogenesis of HIV-1 to get as close as possible to biological significance. We believe that our pooled data-analysis can help estimate the relative contribution of some key genes and pathways of HIV-1 latency and reactivation.

## Concluding remarks and future perspective

To summarize, our pooled data-analysis of different primary CD4^+^ T cell models of HIV-1 latency gave insights into transcriptome profile signatures in latently- and reactivated HIV-1 infected primary CD4^+^ T cells. We identified 130 pdaDEGs in latently- and 117 pdaDEGs in reactivated HIV-1 infected cells from 5 *in vitro* primary CD4^+^ T cell models of HIV-1 latency and 2 *ex vivo* studies of reactivated HIV-1 infected primary CD4^+^ T cells. We observed that in pdaDEGs natural ligands and coreceptors were predominantly downregulated in latently HIV-1 infected primary CD4^+^ T cells, whereas pdaDEGs associated with apoptosis, cell cycle and HLA class II were upregulated in reactivated HIV-1 infected primary CD4^+^ T cells. In addition, we observed 5 pdaDEGs that co-occurred in latently- and reactivated HIV-1 infected primary CD4^+^ T cells, one of which, MSRB2, was found to be differentially expressed between latently- and reactivated HIV-1 infected primary CD4^+^ T cells. This pooled data-analysis is unique in that it analyzes differentially expressed genes of latently- and reactivated HIV-1 infected cells from different *in vitro* primary CD4^+^ T cell models of HIV-1 latency and *ex vivo* studies of reactivated HIV-1 infected primary CD4^+^ T cells from HIV-1 infected individuals, providing insight into differentially expressed genes that might contribute to HIV-1 latency.

## Material and methods

### Study selection for the pooled data-analysis

On the 1^st^ of March 2019 we searched for *in vitro* primary CD4^+^ T cell models of HIV-1 latency on Scopus database. We searched for ((TITLE (HIV OR HIV-1)) AND (TITLE (latent OR latently OR latency)) AND TITLE-ABS-KEY ((transcriptome OR transcriptomics OR “gene expression”))) AND (ALL (“primary cell”)) AND (ALL (“*in vitro* model” OR “cell model”)) AND (LIMIT-TO (DOCTYPE, “ar”)). Each study was independently reviewed and included based on the criterion that each study contained transcriptome data and its methodology derived from an *in vitro* primary CD4^+^ T cell model of HIV-1 latency. In addition, we included two *ex vivo* studies of reactivated HIV-1 infected primary CD4^+^ T cells from HIV-1 infected individuals and our own primary CD4^+^ T cell model of HIV-1 latency.

### Primary CD4^+^ T cells isolation of own model

Detailed description of isolation, transfection and RNAseq data preparation of primary CD4^+^ T cells of own model is given in **Supplementary Material**.

### RNAseq data collection

Raw read counts of Bradley et al. (8), White et al. (11) and Cohn et al. (12). were downloaded from Gene Expression Omnibus (GEO) Database (57). Differentially expressed genes (DEGs) as fold-change were obtained from Kulpa et al. (13) through personal communication. DEGs for Mohammadi et al. (10) were downloaded from the open access interactive web resource http://litchi.labtelenti.org.

### Differential expression testing

All analyses were performed using R software version 4.0.5 (58). We identified DEGs in data sets where raw read counts were available [(8, 10, 12, 13) and our data] using the Bioconductor package EdgeR (59, 60) in a study-by-study basis. Hereby, ambiguous and low-quality reads were removed and genes with low read counts (more than 10 reads in 70% of replicates per group were required) were filtered out. Read counts were normalized *via* trimmed mean of M values (TMM) method. Common dispersion was estimated across all genes by maximizing the negative binomial conditional common likelihood, and tagwise dispersion by an empirical Bayes method based on weighted conditional maximum likelihood. Differential expression testing was performed assuming negative-binomially distributed read counts and computing genewise exact tests for differences in the means between groups. Eventually, fold changes were log2-transformed and p-values adjusted for false discovery rate (FDR) by the Benjamini-Hochberg method.

From Kulpa et al. (13) we selected the RNAseq data for the TCM cell subset, choosing the LRA Bryostatin, as we have seen the least impact on cell viability compared to the other LRAs IL-15 and PMA. As raw read counts were not available and fold changes were given per transcript ID, we filtered for the transcript ID per gene name with the highest CPM. FDR values were estimated using the available p-values with the Bioconductor package qvalue (61).

The microarray data from Iglesias et al. (9) was available only as fold change per gene name, lacking p-values, FDR or raw signals. White et al. (11) previously worked with this dataset and kindly provided us with a dataset comprised of 1297 genes, selected by filtering for adjusted p-value smaller than 0.05.

### Gene annotation and data analysis

Datasets were combined and genes annotations obtained and unified across primary CD4^+^ T cell models of HIV-1 latency using the Bioconductor package bioMart (62, 63) to download annotation Ensembl data (64). HGNC symbol duplications were checked on https://www.genenames.org/tools/multi-symbol-checker/. Known HIV interactions per gene were retrieved from DAVID (Database for Annotation, Visualization and Integrated Discovery) (65, 66) using the HGNC gene symbol and Entrez gene IDs. Functional pathways of genes were obtained from Reactome (67). We calculated the mean standardized expression of genes across all primary CD4^+^ T cell models of HIV-1 latency by averaging over fold changes standardized by study-specific standard error. To account for the differences in the datasets, we refrained from identifying the genes of interest by |fold change| > 2 and FDR < 0.05, but employed a custom filtering method. Hereby a filter score is calculated for every gene based on FDR being smaller than 0.1 and the absolute fold change being greater than the study-specific 50% absolute fold change quantile. Downregulated genes obtain a negative score. Meeting these conditions, a gene can get a score of maximally 2 per study, i.e., maximum score of 8 for latently and 10 for reactivated HIV-1 infected primary CD4^+^ T cells. For each gene the scores are summed up across studies and normalized to 1. Genes of interest were then identified by having a score ≥ 0.5.

### GO enrichment analysis

GO enrichment analysis of genes of interest were performed using the Bioconductor package clusterProfiler (68) using the org.Hs.eg.db Bioconductor annotation package (69) with default settings, apart from setting the minimal size of genes to 3 and choosing a more stringent q-value cutoff of 0.05. Data management and wrangling, as well as visualizations were performed using the R package tidyverse (70). With the parameters set, we could not detect any upregulated biological processes in latently HIV-1 infected CD4^+^ T cells from 4 *in vitro* primary CD4^+^ T cell models of HIV-1 latency.

## Data availability statement

Publicly available datasets were analyzed in this study. This data can be found here (GEO accession number): Bradley et al: SSAMN08685499, SAMN08685500, SAMN08685501, SAMN08685502. White et al: GSE81810; Iglesias-Ussel et al: GSM996242, GSM996243, GSM996244, GSM996245; Mohammadi et al.: http://litchi.labtelenti.org; Kulpa et al. were received upon request.; Cohn et al.: GSM2801437. Inderbitzin et. al. data is available on the European Nucleotide Archive (ENA) of the EMBL’s European Bioinformatics Institute (EMBL-EBI) - Project accession PRJEB53230.

## Author contributions

AI designed the study, performed the literature search, cultured the cells, isolated the RNA for the RNAseq analysis. LO cleaned and performed analysis of the RNAseq datasets. TL downloaded, cleaned and performed analyses of the data sets. PR supported the experiments and analysis of the data. KM supported the analysis of the data, and edited the manuscript. All authors approved the manuscript.

## Funding

This study was funded by the Swiss National Science Foundation, grant No. 310030_141067 and 310030_204404 to KM and from the Forschungskredit Candoc, grant No. FK-19-032 to AI. The funders had no role in study design, data collection and analysis, decision to publish, or preparation of the manuscript.

## Acknowledgments

We want to thank Yik Lim Kok and Roger Kouyos for the fruitful discussion, Catharine Aquino and Andreia Cabral de Gouvea from the Functional Genomics Center Zurich for the RNAseq service.

## Conflict of interest

The authors declare that the research was conducted in the absence of any commercial or financial relationships that could be construed as a potential conflict of interest.

## Publisher’s note

All claims expressed in this article are solely those of the authors and do not necessarily represent those of their affiliated organizations, or those of the publisher, the editors and the reviewers. Any product that may be evaluated in this article, or claim that may be made by its manufacturer, is not guaranteed or endorsed by the publisher.
