# Supplementary material for: Transcriptome profiles of latently- and reactivated HIV-1 infected primary CD4+ T cells: A pooled data-analysis
Source: Front Immunol. 2022 Aug 26;13:915805. doi: 10.3389/fimmu.2022.915805 (PMC9459035; doi:10.3389/fimmu.2022.915805)
Supplement: Supplementary file 1 [file DataSheet_1.docx]

## *Experimental conditions of the 4 in vitro primary CD4^+^ T cell models of HIV-1 latency and 2 ex vivo studies of reactivated HIV-1 infected primary CD4^+^ T cells from HIV-1 infected individuals included in the pooled data-analysis*

The 4 *in vitro* primary CD4^+^ T cell models of HIV-1 latency established different experimental conditions to perform RNA sequencing (RNAseq) or microarray analysis on latently- and reactivated HIV-1 infected cells. In terms of 1. how long cells are maintained in culture prior and after transduction 2. using replication competent or incompetent HIV-1, 3. how CD4^+^ T cell survival is ensured, 4. how reactivation was achieved (supplementary figure 1A). Herein we describe each experimental condition of the 4 *in vitro* primary CD4^+^ T cell models of HIV-1 latency included in our pooled data-analysis.

Iglesias-Ussel *et al*.(1) first isolated primary CD4^+^ T cells and then activated the primary CD4^+^ T cells with anti-CD3 and anti-CD28 antibodies in the presence of IL-2 for 3 days, followed by infection of the cells with a replication-competent HIV-1 (HIV-1_IIIB_). After transduction, the cells were cultured for 10-12 days in medium containing IL-2. To reach a resting state the cells were subsequently cultured in medium containing IL-7. After reaching a resting state, RNA was isolated from sorted p24^gag+^ cells (latently HIV-1 infected primary CD4^+^ T cells) and microarray analysis was performed. The HIV-1 p24^gag+^ antigen synthesized during productive infection remains in the cytoplasm of infected CD4^+^ T cells for several days and slowly decreases during the HIV-1 latency phase. Therefore, detection of p24^gag+^ in the cytoplasm of latently infected cells does not reflect new viral infections. They observed high expression of CD2 in latently HIV-1 infected primary CD4^+^ T cells.

White *et al.*(2) first isolated naïve primary CD4^+^ T cells and then activated the primary CD4^+^ T cells with αCD3/αCD28-coated magnetic beads in the presence of αIL-4, αIL-12, and tumor growth factor (TGF)-β1 for 3 days. After activation, the cells were expanded in medium containing IL-2 for additional 4 days. At day 7 the primary CD4^+^ T cells were transduced with a replication-competent HIV-1 (HIV-1_NL4-3_). After transduction, the cells were cultured with medium containing IL-2 for 3 days and to increase cell-to-cell transmission called “cell crowding” the cells were cultured in round bottom plates in the presence of IL-2 for another 3 days. After that, the cells were cultured in the presence of IL-2 and ART for 4 days. At day 17, cells expressing CD4 were isolated using magnetic bead sorting, with the reasoning that CD4 expression gets downregulated in productively HIV-1 infected cells due to the expression of the accessory genes *Nef* and *Vpu*. At this stage, the cells were considered latently HIV-1 infected primary CD4^+^ T cells. In addition, cell aliquots were subjected to reactivation with αCD3/αCD28-coated magnetic beads in the presence of ART for 2 days. After that, RNA sequencing was performed on latently- and reactivated HIV-1 infected primary CD4^+^ T cells. Analysis after sorting revealed that a number of upregulated p53 related genes in latently infected CD4^+^ T cells were observed in the study of White *et al.* as well as in the study by Iglesias-Ussel *et al*. However, different surface markers on HIV-1 infected versus uninfected CD4^+^ T cells, for instance CD2 observed by Iglesias-Ussel *et al.* (1)*.*, could not be confirmed in White *et al.* (2).

Mohammadi *et al.*(3) first isolated primary CD4^+^ T cells and then activated the primary CD4^+^ T cells with anti-CD3 and anti-CD28 antibodies in the presence of IL-2 for 3 days, followed by infection of the cells with a replication-incompetent HIV-1 GFP reporter virus (NL4-3-Δ6-drEGFP/CXCR4). The replication-incompetent HIV-1 reporter virus uses a CXCR4 tropic HIV envelope for entry. The cells were sorted for GFP expressing cells, 48 hours post transduction. Then the sorted GFP expressing cells were stimulated with anti-CD3 and anti-CD8 antibodies in the presence of IL-2 for ∼3 weeks, to allow cell multiplication. After that, the cells were co-cultured with H80 human brain tumor cell line in the presence of IL-2, to promote survival of primary CD4^+^ T cells, for 10 weeks to reach a resting state. In addition, cell aliquots were subjected to reactivation with T-cell receptor (TCR) stimulation. RNAseq was performed on the latently- and reactivated HIV-1 infected primary CD4^+^ T cells. Cellular transcriptional dynamics was investigated over 10 weeks and according to their model HIV-1 latency appears to be a stable process. They suggest that post-transcriptional blocks also contribute to HIV-1 latency (3). However, viral transcripts were continuously present, accompanied by residual expression of viral-encoded GFP. However, residual GFP or p24 expression was also observed in other primary CD4^+^ T cell models of HIV-1 latency and is interpreted as background (4-8). By using TCR stimulation, they were able to reactivate almost all CD4^+^ T cells shown by increased GFP expression.

Bradley *et al.* (9) first isolated primary CD4^+^ T cells and then activated the primary CD4^+^ T cells with αCD3/αCD28-coated magnetic beads in the presence of IL-2 for 2 days, followed by infection of the cells with a replication-incompetent HIV-1 GFP reporter virus (pNL43-Δ6-dreGFP). The cells were sorted for GFP expressing cells after 2 days post transduction. Then the cells were co-cultured with the H80 human brain tumor cell line in the presence of IL-2 for up to 12 weeks. RNAseq was performed on latently HIV-1 infected primary CD4^+^ T cells. Their main finding was that HIV-1 downregulation occurs in diverse environments but was significantly associated/influenced with the expression of a specific set of host cell genes (9). However, the distribution of CD4^+^ T cells within subpopulations differed from other primary CD4^+^ T cell models of HIV-1 latency (5, 8) which might be explained through the different experimental set-up or the difference of re-stimulation after the first activation done in the study by Tyagi *et al*. (5).

The 2 *ex vivo* studies of reactivated HIV-1 infected primary CD4^+^ T cells from HIV-1 infected individuals used different experimental conditions to investigate HIV-1 infected cells (supplementary figure 1B).

Cohn *et al.* (10) isolated primary CD4+ T cells from ART-suppressed donors and then activated the cells for 36 hours with PHA in the presence of IL-2, 5 antiretroviral drugs (ritonavir, dolutegravir, emtricitabine, tenofovir and maraviroc) and a pan-caspase inhibitor. Then they used the latency capture protocol (LURE) for magnetic enrichment of HIV-1-infected CD4+ T cells using Env-binding broadly neutralising antibodies (biotinylated 3BNC117, 10-1074, PG16), and then the enriched Env-labelled cells were sorted by flow cytometry and single-cell RNAseq was performed. The main finding was that sorted CD4^+^ T cells share a transcriptional profile that includes expression of genes implicated in silencing the virus.

Kulpa *et al*.(11) sorted and performed RNAseq on memory CD4^+^ T_CM_, T_EM_, and T_TM_ subsets from virally suppressed HIV-1 infected individuals, which were stimulated with either PMA/ionomycin, IL-15 or bryostatin for 24h. The major finding was that most intact proviruses are found in effector memory CD4^+^ T cells (11). This finding supports what has already been observed from Hiener *et al.* (12). In addition, the subset distribution was comparable between CD4^+^ T cells of virally suppressed patients and the adapted HIV-1 latency model.

**Material and Methods: Detailed experimental procedure of own model**

## *Primary CD4^+^ T cells isolation*

Primary CD4^+^ T cells were isolated from PBMCs derived from anonymized buffy coats obtained from healthy blood donors as provided by the Blood Donation Service Zurich, Swiss Red Cross, Schlieren, Switzerland. Written informed consent for the use of buffy coats not required for medical treatment for research purposes was obtained from blood donors by the Blood Donation Centre. Enrichment of primary CD4^+^ T cells was performed using the EasySep™ Human CD4^+^ T Cell Enrichment Kit (Miltenyi Biotec). Primary CD4^+^ T cells were kept in culture using central memory media comprising of TexMACS Medium (Miltenyi Biotec) supplemented with 1% Penicillin-Streptomycin (10’000 units/ml Penicillin, 10 mg/ml Streptomycin, Gibco) and 25 ng/mL IL-7, 25 ng/mL IL-15 and 100 U/mL IL-2.

## *Transfection of primary CD4^+^ T cells*

Prior to transfection, the three HIV-1_TRJO.c_ constructs (AAVS1/HIV-1_TRJO.c_ s, *BACH2*/HIV-1_TRJO.c_ c and *NFATC3*/HIV-1_TRJO.c_ c) were linearized using 100 units of PvuI (NEB)/20 µg DNA. For transfection, 5 million enriched primary CD4^+^ T cells were resuspended in 100 μL Nucleofector solution (Human T cell Nucleofector™ Kits, Lonza) and combined with 2 μg of linearized HIV-1_TRJO.c_ plasmid and 2 μg of the corresponding pX458 gRNA/Cas9 plasmid. Nucleofection was performed using the program U-014 for primary cells (Amaxa™ Nucleofector™ II, Lonza). Primary CD4^+^ T cells were cultured in prewarmed culture media. To reach sufficient number of cells, each sample was performed in triplicates. To prevent reinfection 10 μM emtricitabine (nucleoside reverse-transcriptase inhibitor (NRTI)), 5 μM tenofovir (NRTI) and 10 μM dolutegravir (integrase strand transfer inhibitor) were added 4 hrs post infection. At day 9 post transfection, triplicates were combined and sorted for GFP expressing cells using a BD FACSAria™ III (BD Biosciences, Franklin Lakes, NJ, USA). After sorting, RNAseq was performed using Smart-seq2 (13).

## *RNAseq data preparation*

The SUSHI data analysis system (14) was used for RNAseq data analysis for quantitative assessment of gene expression. Within, Kallisto was used for transcript quantification. The data is available on the European Nucleotide Archive (ENA) of the EMBL’s European Bioinformatics Institute (EMBL-EBI) - Project accession PRJEB53230 (15).

**
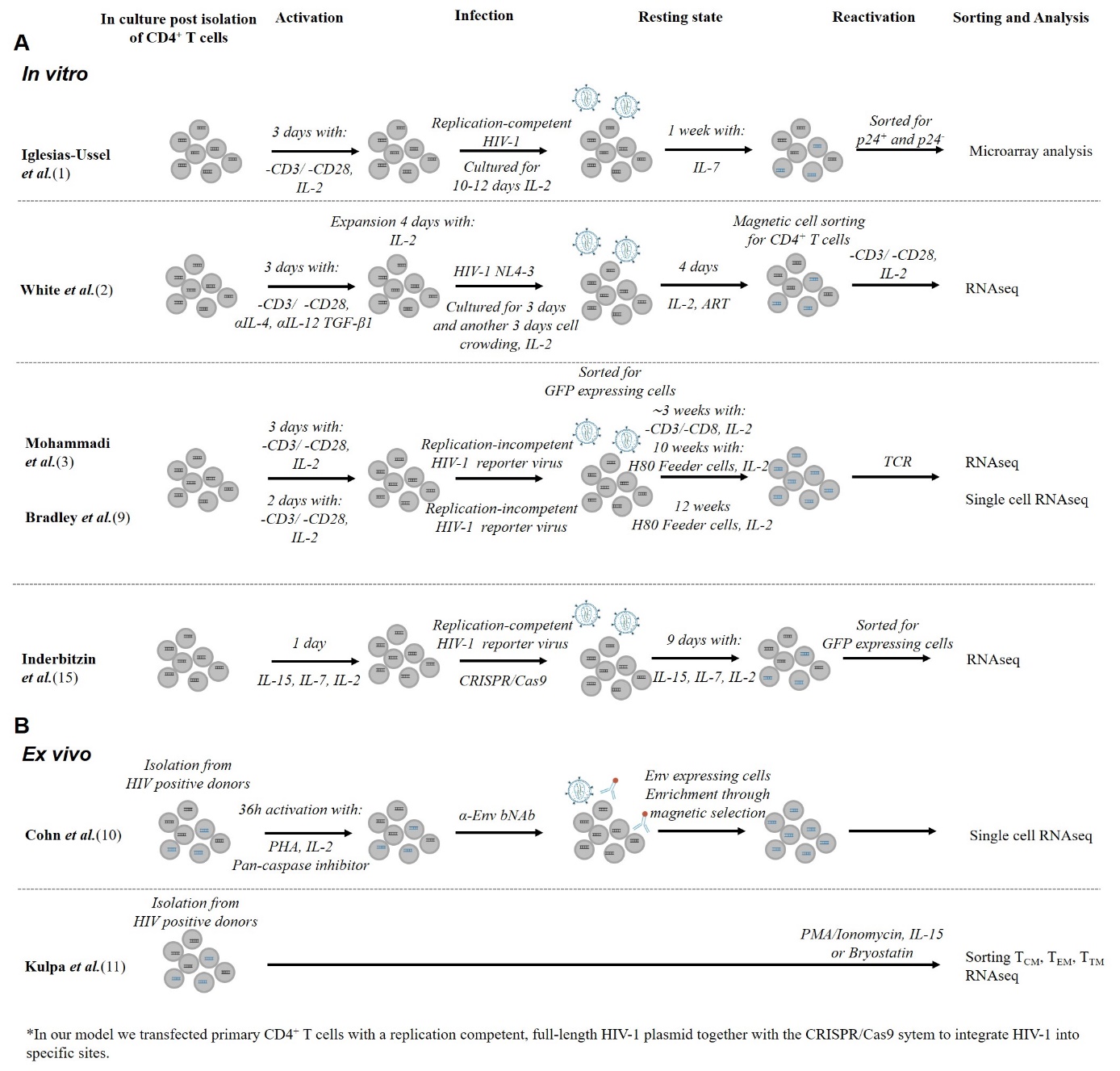
**

**Supplementary figure 1. *In vitro* primary CD4^+^ T cell models of HIV-1 latency and *ex vivo* studies of reactivated HIV-1 infected primary CD4^+^ T cells from HIV-1 infected individuals. A**) Schematic representation of different *in vitro* approaches to sort and RNAseq/microarray analysis of latently- and reactivated HIV-1 infected CD4^+^ T cells. **B**) Schematic representation of different *ex vivo* approaches to sort and RNAseq of reactivated HIV-1 infected CD4^+^ T cells from HIV-1 infected individuals. For each study in **A**)/**B**) the type of activation, infection, resting state, reactivation and type of sorting is depicted.

**Supplementary table 1. Pooled data-analysis differentially expressed genes up- or downregulated in all 4 datasets of latently HIV-1 infected primary CD4^+^ T cells with HIV-1 known associations based on DAVID.**(Envelope (Env), Negative regulatory factor (Nef), Trans-activator of transcription (Tat), Viral protein r (Vpr), regulator of expression of virion proteins (Rev), Viral infectivity factor (Vif) Group-specific antigen (Gag) precursor polyprotein 55 (p55), Polymerase (Pol))

| pdaDEG | up- or downregulated | HIV-1 interaction |
| --- | --- | --- |
| CCL4 (chemokine (C-C motif) ligand 4) | down | Capsid, Env gp120, Env gp160, Env gp41, Nef, Gag p55, Tat, Vpr |
| CCL5 (RANTES) (chemokine (C-C motif) ligand 5) | down | Env gp120, Env gp41, Nef, Gag p55, Tat, Vpr |
| CXCR6 (C-X-C chemokine receptor type 6) | down | Env gp 120 |
| LYZ (lysozyme) | down | Env gp120, Gag-Pol, Nef, Gag p55, Tat |
| RRBP1 (ribosome binding protein 1) | down | Vpr |
| PLAU (plasminogen activator, urokinase) | up | Env gp120, Gag p55, Tat, Vif |
| LMNA (lamin A/C) | up | Rev, Tat, Vpr |
| LY96 (lymphocyte antigen 96) | up | Tat, Vif |
| CD69 (cluster of differentiation 69) | up | Env gp120, Nef, Tat, Vpr |

**Supplementary table 2 Genes up- or downregulated in all 5 datasets of reactivated HIV-1 infected primary CD4^+^ T cells with an HIV-1 known association based on DAVID.** (Envelope (Env), Negative regulatory factor (Nef), Trans-activator of transcription (Tat), Viral protein r (Vpr), Viral protein U (Vpu) Group-specific antigen (Gag) precursor polyprotein 55 (p55), Polymerase (Pol))

| pdaDEG | up- or downregulated | HIV-1 interaction |
| --- | --- | --- |
| ACTA2 (actin alpha 2, smooth muscle) | up | Env gp120, Env gp160, Env p41, matrix, Nef, nucleocapsid, Gag p55, retropepsin Tat, Vpr |
| CXCL10 (C-X-C motif chemokine ligand 10) | up | Capsid, Env gp120, Env gp41, Nef, Tat, Vpr |
| HLA-DOA (major histocompatibility complex, class II, DO alpha) | up | Env gp120, Env gp160, Env gp41, Nef, Gag p55, Tat, Vpu |
| LAMP3 (lysosomal associated membrane protein 3) | up | Env gp120, Tat |
| SLC7A11 (solute carrier family 7 member 11) | up | Tat |
| SPTBN5 (spectrin beta, non-erythrocytic 5) | up | retropepsin |

# References

1. Iglesias-Ussel M, Vandergeeten C, Marchionni L, Chomont N, Romerio F. High Levels of CD2 Expression Identify HIV-1 Latently Infected Resting Memory CD4+ T Cells in Virally Suppressed Subjects. *J Virol* (2013) 87(16):9148-58. Epub 2013/06/14. doi: 10.1128/jvi.01297-13.

2. White CH, Moesker B, Beliakova-Bethell N, Martins LJ, Spina CA, Margolis DM, et al. Transcriptomic Analysis Implicates the p53 Signaling Pathway in the Establishment of Hiv-1 Latency in Central Memory CD4 T Cells in an in Vitro Model. *PLoS Pathog* (2016) 12(11):e1006026. Epub 2016/11/30. doi: 10.1371/journal.ppat.1006026.

3. Mohammadi P, di Iulio J, Munoz M, Martinez R, Bartha I, Cavassini M, et al. Dynamics of HIV Latency and Reactivation in a Primary CD4+ T Cell Model. *PLoS Pathog* (2014) 10(5):e1004156. Epub 2014/05/31. doi: 10.1371/journal.ppat.1004156.

4. Sahu GK, Lee K, Ji J, Braciale V, Baron S, Cloyd MW. A Novel in Vitro System to Generate and Study Latently HIV-Infected Long-Lived Normal CD4+ T-Lymphocytes. *Virology* (2006) 355(2):127-37. Epub 2006/08/22. doi: 10.1016/j.virol.2006.07.020.

5. Tyagi M, Pearson RJ, Karn J. Establishment of HIV Latency in Primary CD4+ Cells Is Due to Epigenetic Transcriptional Silencing and P-TEFb Restriction. *J Virol* (2010) 84(13):6425-37. Epub 2010/04/23. doi: 10.1128/jvi.01519-09.

6. Marini A, Harper JM, Romerio F. An in Vitro System to Model the Establishment and Reactivation of HIV-1 Latency. *J Immunol* (2008) 181(11):7713-20. Epub 2008/11/20. doi: 10.4049/jimmunol.181.11.7713.

7. Bosque A, Planelles V. Induction of HIV-1 Latency and Reactivation in Primary Memory CD4+ T Cells. *Blood* (2009) 113(1):58-65. Epub 2008/10/14. doi: 10.1182/blood-2008-07-168393.

8. Yang HC, Xing S, Shan L, O'Connell K, Dinoso J, Shen A, et al. Small-Molecule Screening Using a Human Primary Cell Model of HIV Latency Identifies Compounds That Reverse Latency without Cellular Activation. *J Clin Invest* (2009) 119(11):3473-86. Epub 2009/10/07. doi: 10.1172/jci39199.

9. Bradley T, Ferrari G, Haynes BF, Margolis DM, Browne EP. Single-Cell Analysis of Quiescent Hiv Infection Reveals Host Transcriptional Profiles That Regulate Proviral Latency. *Cell Rep* (2018) 25(1):107-17.e3. Epub 2018/10/04. doi: 10.1016/j.celrep.2018.09.020.

10. Cohn LB, da Silva IT, Valieris R, Huang AS, Lorenzi JCC, Cohen YZ, et al. Clonal CD4(+) T Cells in the HIV-1 Latent Reservoir Display a Distinct Gene Profile Upon Reactivation. *Nat Med* (2018) 24(5):604-9. Epub 2018/04/25. doi: 10.1038/s41591-018-0017-7.

11. Kulpa DA, Talla A, Brehm JH, Ribeiro SP, Yuan S, Bebin-Blackwell AG, et al. Differentiation into an Effector Memory Phenotype Potentiates HIV-1 Latency Reversal in CD4(+) T Cells. *J Virol* (2019) 93(24). Epub 2019/10/04. doi: 10.1128/jvi.00969-19.

12. Hiener B, Horsburgh BA, Eden JS, Barton K, Schlub TE, Lee E, et al. Identification of Genetically Intact HIV-1 Proviruses in Specific CD4(+) T Cells from Effectively Treated Participants. *Cell Rep* (2017) 21(3):813-22. Epub 2017/10/19. doi: 10.1016/j.celrep.2017.09.081.

13. Picelli S, Faridani OR, Björklund AK, Winberg G, Sagasser S, Sandberg R. Full-Length RNA-Seq from Single Cells Using Smart-Seq2. *Nat Protoc* (2014) 9(1):171-81. Epub 2014/01/05. doi: 10.1038/nprot.2014.006.

14. Hatakeyama M, Opitz L, Russo G, Qi W, Schlapbach R, Rehrauer H. Sushi: An Exquisite Recipe for Fully Documented, Reproducible and Reusable Ngs Data Analysis. *BMC Bioinformatics* (2016) 17(1):228. doi: 10.1186/s12859-016-1104-8.

15. Inderbitzin A, Loosli, T., Opitz, L., Rusert, P., Metzner, K.J. Transcriptomic Analysis Showed Upregulation of Genes Associated with p53- and Pi3k/Akt Pathway in Primary CD4+ T Cells after CRISPR/Cas9-Mediated Insertion of Replication-Competent HIV-1 into Three Target Sites (Unpublished Data), Project Accession PRJEB53230 *EMBL’s European Bioinformatics Institute (EMBL-EBI)* (2022).
